# Phosphorylated ERK is a potential predictor of sensitivity to sorafenib when treating hepatocellular carcinoma: evidence from an *in vitro *study

**DOI:** 10.1186/1741-7015-7-41

**Published:** 2009-08-24

**Authors:** Zhe Zhang, Xiaoyun Zhou, Hujia Shen, Dexing Wang, Yanhong Wang

**Affiliations:** 1 Liver Cancer Institute, Zhongshan Hospital, Fudan University, Shanghai 200032, PR China

## Abstract

**Background:**

Sorafenib is the first agent that has demonstrated an improved overall survival benefit in advanced hepatocellular carcinoma (HCC), setting a new standard for first-line treatment. However, no one has yet been able to predict sensitivity to sorafenib. Pre-treatment pERK level has been shown to be associated with favorable response to such therapy in a phase II clinical study, indicating that pERK may be a potential biomarker for treatment of HCC with sorafenib.

**Methods:**

The effects of sorafenib and 5-fluorouracil (5-FU) on cell proliferation were evaluated by cell viability assays in four HCC cell lines (SMMC-7721, MHCC97-L, MHCC97-H and HCCLM6) with different metastatic potential and basal pERK expression levels. Expression levels of pERK were determined by immunocytochemical quantification together with western blot analysis, and pERK density values were also calculated. Correlation analyses were then carried out between the IC_50 _values of drugs and pERK density values. After basal ERK phosphorylation was down-regulated with U0126 in MHCC97-H cells, cellular responsiveness to sorafenib was assessed by cell viability assay.

**Results:**

Basal pERK levels increased stepwise in cell lines in accordance with their metastatic potential. Sorafenib inhibited ERK phosphorylation in a dose-dependent manner in all four cell lines at a concentration between 5 and 20 μM, but the degree of inhibition was significantly different according to their basal pERK expression level (*P *< 0.0001). In contrast, no significant change was observed after 5-FU treatment. Correlation analyses between the IC_50 _values and pERK densities revealed that the effects of sorafenib on cell proliferation were significantly correlated with basal pERK levels (Spearman r = -0.8671, *P *= 0.0003). Resistance to 5-FU was also significantly associated with basal pERK expression in these HCC cell lines (Spearman r = 0.7832, *P *= 0.0026). After the basal ERK phosphorylation level in MHCC97-H cells was reduced with U0126, they were significantly less sensitive to sorafenib-mediated growth inhibition, with an IC_50 _of 17.31 ± 1.62 μM versus 10.81 ± 1.24 μM (*P *= 0.0281).

**Conclusion:**

In this *in vitro *study, pERK was confirmed to be a potential biomarker predictive of sensitivity to sorafenib in treating HCC. The RAF/MEK/ERK pathway may be involved in drug resistance to traditional chemotherapy in HCC.

## Background

Hepatocellular carcinoma (HCC) is the sixth most common malignancy worldwide and the third most common cause of death from cancer, accounting for more than 626,000 new cases and 598,000 deaths per year. Of all these cases, more than half are in China alone [1]. The disease is diagnosed at early stages in 30 to 40% of all patients and is amenable to potentially curative treatments, such as surgical therapies (resection and liver transplantation) and locoregional procedures (radiofrequency ablation). Five-year survival rates of up to 60 to 70% can be achieved in well-selected patients [2]. However, disease diagnosed at an advanced stage or with progression after locoregional therapy has a dismal prognosis, owing to the underlying liver disease and lack of effective treatment options [3]. No systemic therapy with traditional chemotherapy drugs has improved survival in patients with advanced hepatocellular carcinoma [4].

Sorafenib (Nexavar, Bayer HealthCare Pharmaceuticals) is an oral multikinase inhibitor that inhibits the serine-threonine kinases Raf-1 and B-Raf, the receptor tyrosine kinase activity of vascular endothelial growth factor (VEGF) receptors 1, 2, and 3, and platelet-derived growth factor receptor β [5]. It blocks tumor cell proliferation and tumor angiogenesis, and increases the rate of apoptosis in a wide range of tumor models by targeting the Raf/mitogen-activated protein kinase kinase/extracellular signal-regulated kinase (RAF/MEK/ERK) and VEGF signaling pathways [6]. The results of a phase III, randomized, placebo-controlled trial, the Sorafenib HCC Assessment Randomized Protocol (SHARP) trial, were recently presented [7]. In this trial, sorafenib demonstrated improved overall survival and time to tumor progression in patients with advanced HCC. This landmark study represents the first agent that has demonstrated an improved overall survival benefit in this disease and sets a new standard for the first-line treatment of advanced HCC that has been approved by the US Food and Drug Administration (FDA).

However, no one has yet predicted sensitivity to sorafenib in the treatment of HCC. It is well known that phosphorylated ERK (pERK) is a key downstream component of the RAF/MEK/ERK signaling pathway. It can be translocated to the nucleus after phosphorylation, where it leads to changes in gene expression by phosphorylating and regulating various transcription factors, such as Ets family transcription factors (for example, Elk-1) [8]. In a phase II study in 137 patients with advanced, inoperable HCC, of which 33 had their pre-treatment pERK levels evaluated, pre-treatment tumor pERK levels were correlated with the time to tumor progression. Patients whose tumors expressed higher baseline pERK levels had a longer time to tumor progression following treatment with sorafenib [9]. These data suggest that tumors containing higher levels of pERK are more sensitive, or responsive, to sorafenib, indicating that pERK may be a useful biomarker in treating HCC with sorafenib. Whether this marker will prove to be predictive of response needs to be validated in future studies.

To investigate the relationship between the effects of sorafenib on cell proliferation and basal pERK levels in HCC cell lines, here we evaluate the effects of sorafenib on four HCC tumor cell lines (SMMC-7721, MHCC97-L, MHCC97-H and HCCLM6) with different metastatic potentials and baseline pERK expression levels. A series of human HCC cell lines with similar genetic backgrounds yet dramatic differences in spontaneous metastatic behaviors, which had been established at the authors' institute [10,11], provided a unique platform for this research. Among these cell lines, SMMC-7721 is low-invasive and non-metastatic. MHCC97-L and MHCC97-H are two different metastatic HCC cell clones isolated from the same parent cell line MHCC97, which was derived from a nude mouse model of human HCC metastasis (LCI-D20). The LCI-D20 model was developed by orthotopic inoculation of an intact tumour tissue of an intrahepatic disseminated lesion from a 39-year-old Chinese male patient with HCC (Zhongshan Hospital, Fudan University, Shanghai, China) in whose serum abnormal alpha-fetoprotein and HBsAg (hepatitis B surface antigen) were found. Spontaneous pulmonary metastasis occurred in 40% and 100% of recipient nude mice after orthotopic transplantation of MHCC97-L and MHCC97-H, respectively. HCCLM6 was established from MHCC97-H by six rounds of *in vivo *metastasis selection and produced further multiple extensive metastases through both blood vessels and lymphatic channels. Such characteristics make these cell lines valuable for comparative study.

## Materials and methods

### Drug preparations

Sorafenib tosylate (Nexavar, [N-(3-trifluoromethyl-4-chlorophenyl)-N-(4-(2-methylcarbamoylyridin-4-yl)oxyphenyl)urea]) was a gift from Bayer Schering Phama. The MEK1/2 inhibitor U0126 was purchased from Cell Signaling Technologies Inc. (Beverly, MA, USA). Sorafenib and U0126 were dissolved in 100% dimethyl sulfoxide (DMSO; Sigma, St Louis, MO, USA) and diluted with RPMI 1640 or Dulbecco's modified Eagle's medium (DMEM) to the desired concentration with a final DMSO concentration of 0.1% (v/v) for *in vitro *studies. DMSO was added to cultures at 0.1% (v/v) as a solvent control. Fluorouracil injection was purchased from Shanghai Xudong Haipu Pharmaceutical Co., Ltd (Shanghai, China) and was diluted directly with cell culture media to the desired concentration.

### Cell lines

SMMC-7721 human HCC tumor cells were obtained from the Institute of Biochemistry and Cell Biology, Shanghai Institutes for Biological Sciences, Chinese Academy of Sciences (Shanghai, China) and cultured in RPMI 1640. MHCC97-L, MHCC97-H and HCCLM6 human HCC tumor cells were obtained from the Liver Cancer Institute of Fudan University (Shanghai, China) and cultured in DMEM. All cells were cultured at 37°C in 5% CO_2 _in culture media containing 10% fetal bovine serum. Unless otherwise indicated, cell culture reagents were purchased from GIBCO BRL Company (Grand Island, NE, USA).

### Immunocytochemical staining and quantification

Cells were plated in six-well plates with cover slips at 4 × 10^5 ^per well. On the following day, cells were treated with compounds indicated in the experiment. Briefly, cells were exposed to 5, 10, or 20 μM sorafenib for 24 hours. Cells were exposed to 20 μM U0126 for 6 hours. DMSO was added to cultures at 0.1% (v/v) as a solvent control. Cells were treated with 10, 20, or 50 mg/l 5-fluorouracil (5-FU) for 48 hours. Cell culture medium without 5-FU was used as a control. After being fixed in acetone and blocked serially with IHC Biotin Block kit, 3% H_2_O_2_, and 10% normal goat serum, sections were incubated with the mouse monoclonal antibody to ERK1 + ERK2 (Clone MAPK-YT, Abcam, Cambridge, MA, USA) at 1:100 dilution overnight at 4°C. The UltraSensitive™ S-P stain system (Maixin Biotechnology Development Co., Ltd, Fuzhou, China) was applied according to the manufacturer's instructions. Sections were then developed in diaminobenzidine solution and counterstained with Mayer's hematoxylin. Negative controls were performed by omitting the primary antibodies.

Sections were observed at 200× magnification in a computerized image system composed of a Leica CCD camera DFC420 connected to a Leica DM IRE2 microscope (Leica Microsystems Imaging Solutions Ltd, Cambridge, United Kingdom) and images were captured by the Leica QWin Plus version 3 software under the same conditions. The same protein quantification method was used for pERK quantification with Image-Pro Plus version 6.2 software (Media Cybernetics Inc., Bethesda, MD, USA) as Sun's group reported [12]. The pERK density in each field was calculated as [Integrated optical density of pERK-positive objects/(Total field area – Blank area)]. The mean value of pERK density in each group was calculated on six random field samples from three independent experiments with replicates per experiment. The expression rate of pERK was calculated from pERK density and the expression rate in the control group of each cell line was set as the 100% baseline. Data within each group were analyzed statistically with one-way ANOVA and differences between cell lines of sorafenib's pERK inhibition were analyzed by two-way ANOVA, both of which were followed by Bonferroni's multiple comparison test with SPSS 13.0 for Windows (SPSS Inc. Chicago, IL, USA). *P *< 0.05 was considered significant.

### Immunoblot analysis

Cells were plated at 6 × 10^5 ^cells per well in six-well plates. On the following day, cells were treated with the same methods as described above. After treatment, cells were washed with cold phosphate-buffered saline and lysed using RIPA lysis buffer containing 1 mM phenylmethylsulfonyl fluoride (PMSF). Twenty micrograms of protein, which was determined using a bicinchoninic acid protein assay, from control and treated cell lysates was loaded on 5% and 12% SDS-PAGE gels, electrophoresed at a constant voltage of 70 V for 2 hours, and transferred onto PVDF membranes (0.45 μm) at a constant voltage of 80 V for 2.5 hours. Blots were probed with a 1:1,000 dilution of mouse monoclonal to ERK1 + ERK2 antibody (Clone MAPK-YT, Abcam, Cambridge, MA, USA), a 1:3,000 dilution of anti-human β-actin monoclonal antibody, then horseradish peroxidase-conjugated secondary antibody (1:500) and detected by enhanced chemiluminescence reagent (ECL kit, Pierce, Rockford, IL, USA). Unless otherwise indicated, immunoblot reagents were purchased from Beyotime Institute of Biotechnology (Shanghai, China).

### Cell viability assay

Cells were plated at 5,000 cells per well in 96-well microtiter plates and incubated overnight at 37°C in a humidified incubator containing 5% CO_2_. On the following day, compounds were added to the wells indicated in the experiment. Cells were exposed to sorafenib for 24 hours at concentrations of 0.01, 0.1, 1, 2, 4, 5, 10, 15, 20, 25 or 30 μM, and to U0126 for 6 hours at concentrations of 1, 5, 10, 20, 50 or 100 μM. In the sequential combination experiment, cells were pretreated with 20 μM U0126 for 6 hours and then exposed to sorafenib for a further 24 hours. DMSO was added to cultures at 0.1% (v/v) as a solvent control. Cells were treated with 5-FU for 48 hours at concentrations of 0.01, 0.1, 1, 5, 10, 20, 50, 100, 200, 500 or 1,000 mg/l. Cell culture medium without 5-FU was used as a control. Cell viability was determined using the Cell Counting Kit-8 (Dojindo Laboratories, Kumamoto, Japan) according to the manufacturer's instructions [13]. IC_50 _values were calculated by nonlinear regression analysis using GraphPad Prism version 5.0 software (GraphPad Software, Inc., San Diego, CA, USA) [6], according to the results of at least three independent experiments with four replicates of each cell line per experiment. Differences in cellular responsiveness to drugs were analyzed statistically with two-way ANOVA with SPSS 13.0 for Windows (SPSS Inc.). Spearman's rank correlation method was used for correlation analyses between pERK density values and drugs' IC_50 _values of three independent experiments for four cell lines with four replicates each (SPSS 13.0 for Windows, SPSS Inc.). *P *< 0.05 was considered significant.

## Results

### Basal pERK levels in HCC cell lines increase stepwise with their metastatic potential

**Figure 1:**
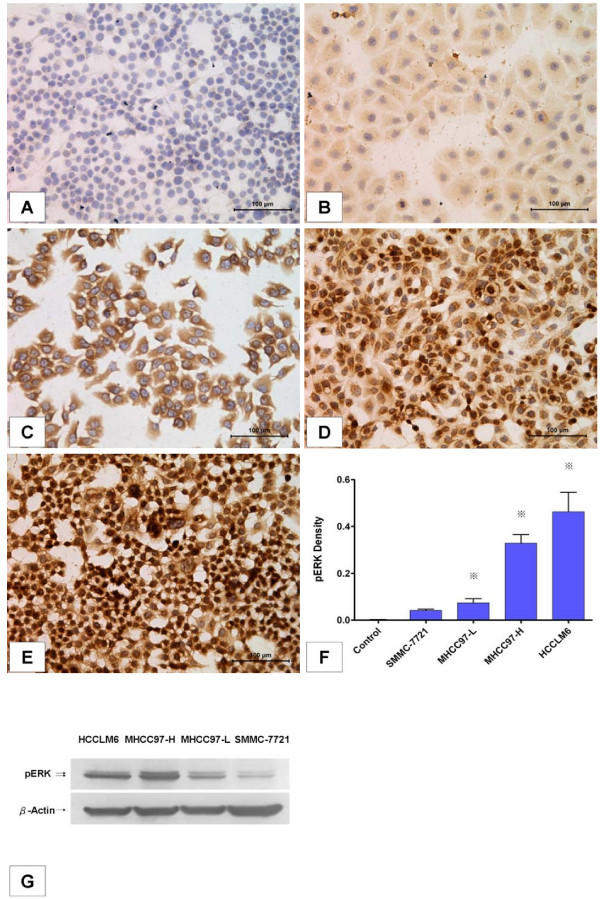
**Basal pERK levels in hepatocellular carcinoma (HCC) cell lines increased stepwise in accordance with their metastatic potential**. **(A-E) **Immunocytochemical staining of pERK (200×). (A) Negative control. (B) SMMC-7721. (C) MHCC97-L. (D) MHCC97-H. (E) HCCLM6. **(F) **Quantification of basal pERK levels in different HCC cell lines. pERK density was quantified using Image-Pro Plus version 6.2 software as described in Materials and methods. Columns represent means of six samples in each group; bars indicate standard deviation; *, *P *< 0.05, when compared to the negative control. **(G) **Western blot analysis of pERK protein in different HCC cell lines.

Basal pERK levels in four HCC cell lines were measured by immunocytochemistry and image quantification. Immunocytochemical analysis showed that pERK proteins were found in both the nuclei and cytoplasm of tumor cells. However, pERK in cell lines with higher metastatic potential seemed more inclined to be located in the nucleus, with stronger staining intensity (Figure 1B–E). The results of image quantification confirmed that baseline pERK was differentially expressed in these HCC cell lines (*P *< 0.0001, n = 6) and seemed to be correlated with their metastatic potential. The pERK density in SMMC-7721, MHCC97-L, MHCC97-H and HCCLM6 cells was 0.042 ± 0.006, 0.081 ± 0.007, 0.329 ± 0.037 and 0.463 ± 0.084, respectively. In metastatic MHCC97-H and HCCLM6 cells, pERK levels were significantly higher than in non-metastatic SMMC-7721 cells (*P *< 0.0001, n = 6). Even among the three metastatic cell lines, pERK levels were differentially expressed and increased stepwise with their metastatic potential (*P *< 0.0001, n = 6; Figure 1F).

Baseline ERK phosphorylation levels in these cancer cells were also examined by western blot analysis. Consistent with immunocytochemical analysis, the results demonstrated that cancer cells with more invasive potential such as HCCLM6 and MHCC97-H cells expressed higher levels of pERK when compared to the relatively less invasive MHCC97-L or SMMC-7721 cells (Figure 1G).

### Effects of sorafenib on ERK phosphorylation inhibition are significantly associated with basal pERK levels in HCC cell lines

**Figure 2:**
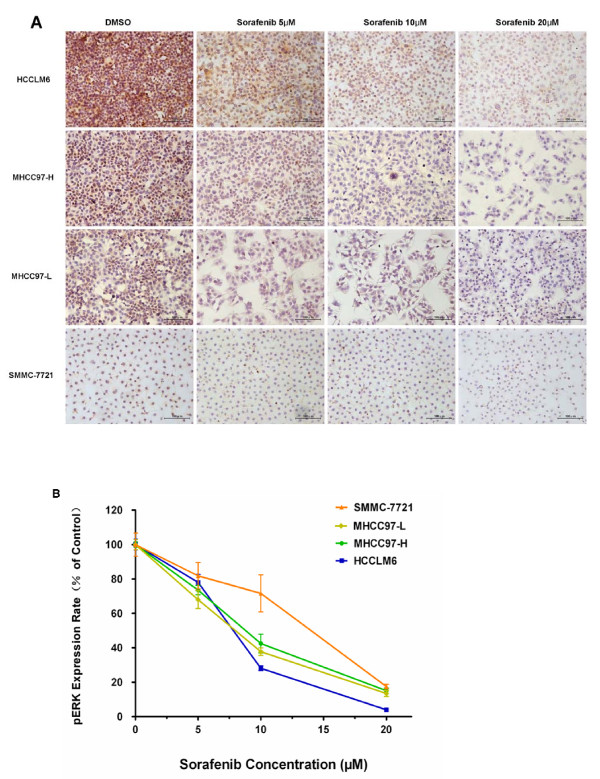
**The degree of sorafenib's inhibition of ERK phosphorylation was significantly associated with basal pERK levels in hepatocellular carcinoma (HCC) cell lines**. **(A) **Immunocytochemical staining of pERK (200×) after treatment with sorafenib for 24 hours in four HCC cell lines. **(B) **pERK expression rate in HCC cell lines after sorafenib treatment. pERK levels were reduced after sorafenib treatment in all four cell lines, but the inhibition effect in SMMC-7721 cells, with lower levels of pERK, was significantly weaker when compared to the other three HCC cell lines with relatively higher basal pERK levels (two-way ANOVA, *P *< 0.0001, n = 6). The expression rate of pERK was calculated from pERK density determined as described in Materials and methods and the rate in each control group was set as the 100% baseline. Columns represent means of six samples in each group; bars indicate standard error.

The pERK protein is best known as a key downstream component of the RAF/MEK/ERK pathway. Changes in the levels of ERK phosphorylation were determined by immunocytochemical analysis in order to evaluate the effects of sorafenib on this pathway. In our study, sorafenib could inhibit ERK phosphorylation in all four HCC cell lines dose-dependently at a concentration between 5 and 20 μM (Figure 2A). After exposure to 5, 10 or 20 μM sorafenib for 24 hours, the expression rate of pERK in SMMC-7721 cells fell gradually to 81.88 ± 7.65%, 71.63 ± 10.80% and 17.47 ± 1.34%, respectively, and in HCCLM6 cells to 78.06 ± 4.66%, 28.12 ± 1.36% and 3.99 ± 0.19%, respectively (Figure 2B). The expression rates in both cell lines were significantly reduced when compared to each DMSO control group (*P *= 0.0043, n = 6, and *P *< 0.0001, n = 6, respectively). However, further statistical analyses revealed the significant difference in the degree of the sorafenib effects in these HCC cell lines. Interestingly, the sorafenib pERK inhibition effect in SMMC-7721 cells with lower initial levels of pERK was significantly weaker when compared to the other three HCC cell lines with relatively higher basal pERK levels (*P *< 0.0001, n = 6; Figure 2B), and it should be noted that this difference was mainly at 10 μM sorafenib. No significant difference was found in MHCC97-L, MHCC97-H and HCCLM6 cells.

**Figure 3:**
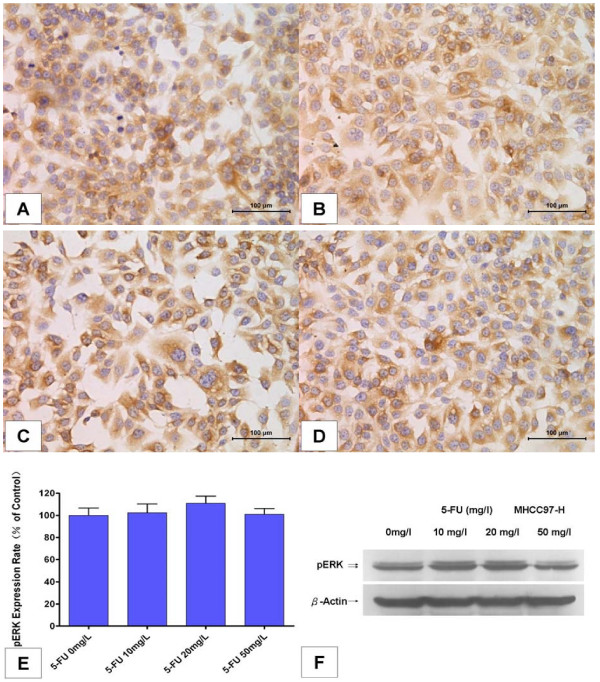
**5-Fluorouracil (5-FU) hardly inhibited ERK phosphorylation in MHCC97-H cells**. **(A-D) **Immunocytochemical staining of pERK (200×) after treatment with 5-FU for 48 hours in MHCC97-H cells. (A) Culture medium without 5-FU was used as control. (B) 5-FU 10 mg/l. (C) 5-FU 20 mg/l. (D) 5-FU 50 mg/l. **(E) **pERK expression was not reduced after 5-FU treatment. The expression rate of pERK was calculated from pERK density determined as described in Materials and methods and the rate in the control group was set as the 100% baseline. Columns represenr means of six samples in each group; bars indicate standard deviation; *, *P *< 0.05, when compared to the control. **(F) **Western blot analysis of pERK protein in MHCC97-H cells after treatment with 5-FU for 48 hours.

On the contrary, no significant change was observed after 5-FU treatment in MHCC97-H cells (Figure 3A–E). The pERK expression rate was 102.3 ± 7.88%, 110.8 ± 6.60%, and 101.1 ± 5.12%, respectively, after exposure to 10, 20 or 50 mg/l 5-FU for 48 hours, with no statistical difference with the control group (*P *> 0.05, n = 6; Figure 3E). Western blot analysis confirmed the same results above (Figure 3F).

### Effects of sorafenib on cell proliferation are significantly correlated with basal pERK levels in HCC cell lines

**Figure 4:**
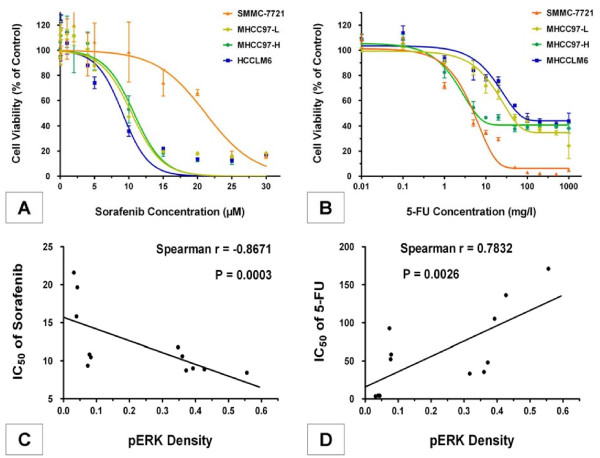
**Effects of sorafenib and resistance to 5-fluorouracil (5-FU) were significantly correlated with basal pERK levels in hepatocellular carcinoma (HCC) cell lines**. **(A) **Sorafenib inhibited cell proliferation in different HCC cell lines. **(B) **5-FU inhibited cell proliferation in different HCC cell lines. Each value represents the average of four independent determinations with four replicates per experiment. Bars indicate standard error. **(C) **Correlation analysis between the IC_50 _values of sorafenib and pERK density values. **(D) **Correlation analysis between the IC_50 _values of 5-FU and pERK density values. The IC_50 _value, at which 50% of cell growth is inhibited compared with control, was calculated by nonlinear regression analysis using GraphPad Prism version 5.0 software. pERK density was quantified using Image-Pro Plus version 6.2 software as described in Materials and methods. Spearman's rank correlation method was used for correlation analysis between pERK density values and drugs' IC_50 _values of three independent experiments for four cell lines with four replicates each. *P *< 0.05 was considered significant.

The effects of sorafenib on cell proliferation were measured by the CCK-8 cell viability assay. According to our results, sorafenib inhibited proliferation of all four HCC cell lines in a dose-dependent manner as described in previous research [6], with an IC_50 _of 20.85 ± 2.81 μM, 10.38 ± 1.52 μM, 10.70 ± 2.35 μM and 9.11 ± 2.44 μM in SMMC-7721, MHCC97-L, MHCC97-H and HCCLM6 cells, respectively. As expected, SMMC-7721 cells, which contain the lowest levels of pERK, were significantly less sensitive to sorafenib-mediated growth inhibition than the other three HCC cell lines with higher basal pERK levels (*P *= 0.0018, n = 4; Figure 4A). Meanwhile, a significant negative correlation (Spearman r = -0.8671, *P *= 0.0003, n = 12) was observed between the IC_50 _values of sorafenib in these HCC cell lines and their pERK density values (Figure 4C), indicating that the effects of sorafenib on cell proliferation were significantly correlated with basal pERK levels in these HCC cell lines.

Opposite results were observed with treatment with the traditional chemotherapy drug 5-FU. 5-FU inhibited HCC cell proliferation with an IC_50 _of 4.24 ± 0.87 mg/l, 79.71 ± 24.49 mg/l, 41.21 ± 21.55 mg/l and 187.45 ± 78.05 mg/l in SMMC-7721, MHCC97-L, MHCC97-H and HCCLM6 cells, respectively, with significant statistical differences (*P *< 0.0001, n = 4). The SMMC-7721 cells, with lower pERK expression, demonstrated a higher sensitivity to 5-FU. However, MHCC97-L, MHCC97-H, and HCCLM6 cells, with higher pERK expression, exhibited more resistance to this drug. The ultimate inhibition rate before reaching a plateau in these three cell lines was about 35%, 40%, and 45%, respectively, each compared to its control group (Figure 4B). Furthermore, a significant correlation (Spearman r = 0.7832, *P *= 0.0026, n = 12) was observed between the IC_50 _values of 5-FU and pERK density values (Figure 4D), indicating that the resistance to 5-FU was significantly associated with basal pERK expression in these HCC cell lines.

### Effects of MEK/ERK pathway inhibition and pERK reduction on sensitivity to sorafenib

**Figure 5:**
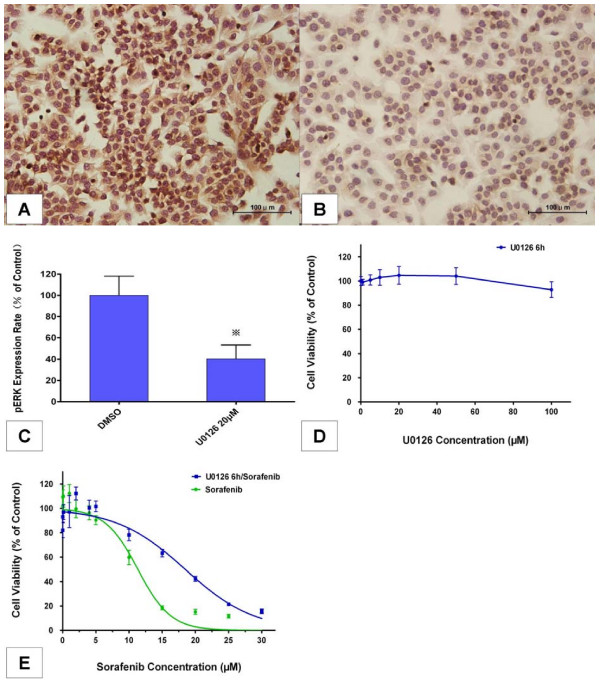
**Effects of reducing basal pERK levels on sorafenib sensitivity**. **(A, B)** Immunocytochemical staining of pERK (200×) in MHCC97-H cells after treatment with 20 μM U0126 for 6 hours. (A) DMSO (0.1%) was used as solvent control. (B) U0126 20 μM. **(C) **pERK expression was reduced after U0126 treatment in MHCC97-H cells. The expression rate of pERK was calculated from pERK density determined as described in Materials and methods and the rate in the control group was set as the 100% baseline. Columns represent means from six samples in each group; bars indicate standard deviation; *, *P *< 0.05, Student's *t*-test was used when compared with solvent control. **(D) **Effects of U0126 treatment for 6 hours on cell proliferation in MHCC97-H cells. **(E) **Effects of sorafenib individually and in sequential combination with U0126 on cell proliferation in MHCC97-H cells. In the sequential combination experiments, cells were pre-treated with 20 μM U0126 for 6 hours and then exposed to sorafenib for a further 24 hours. Each value represents the average of six independent determinations with four replicates per experiment; bars indicate standard error.

To more directly determine the relationship between pERK expression and sensitivity to sorafenib, we inhibited the MEK/ERK pathway and reduced basal pERK expression in MHCC97-H cells via U0126, a selective inhibitor of MEK 1 and MEK 2 [14], and then compared cellular responsiveness to sorafenib to that of untreated cells. To avoid possible direct growth inhibition by U0126, exposure of cells to this drug was limited to 6 hours according to our preliminary experiments. Quantification of cellular pERK levels by immunocytochemical analysis indicated that constitutive ERK phosphorylation was strongly reduced in MHCC97-H cells after treatment with 20 μM U0126 for 6 hours, relative to the level observed in the untreated cells (60% inhibition; Figure 5A–C), which induced almost no detectable systemic toxicity on cell proliferation (Figure 5D).

In the following experiments, we compared sorafenib responsiveness of MHCC97-H cells pretreated with 20 μM U0126 for 6 hours to an untreated control. Cell viability assay revealed that the pretreated cells were significantly less sensitive to sorafenib-mediated growth inhibition, with an IC_50 _of 17.31 ± 1.62 μM versus 10.81 ± 1.24 μM (*P *= 0.0281, n = 6; Figure 5E). These results confirmed that the RAF/MEK/ERK signaling pathway was essential for sorafenib-mediated growth inhibition, and that the sensitivity to sorafenib was directly related to the activation of this pathway and basal pERK expression in MHCC97-H cells.

## Discussion

It is well known that the RAF/MEK/ERK cascade is a key signaling pathway involved in the regulation of normal mammalian cell proliferation, survival and differentiation. It couples signals from cell surface receptors to transcription factors and regulates gene expression though a phosphorylation cascade. Raf serine/threonine kinases phosphorylate and activate the MEK1/2 dual-specificity protein kinases, which then phosphorylate and activate ERK1/2. Activated ERK is a downstream component of an evolutionarily conserved signaling module that can be translocated to the nucleus, where it phosphorylates and regulates various transcription factors, ultimately leading to changes in gene expression. Additionally, Ras family small GTPases are key upstream activators of the RAF/MEK/ERK pathway, which are often activated by upstream molecules such as receptor tyrosine kinases (for example, epidermal growth factor receptor, VEGF receptor and transforming growth factor-α receptors) [8]. Mutation or over-activation of related components in the RAF/MEK/ERK cascade would lead to acceleration of cell proliferation and extension of survival, thus contributing to human oncogenesis [15].

This pathway has been implicated in the molecular pathogenesis of HCC. First of all, as an upstream activator of this pathway, the *Ras *gene is mutationally activated in 30% of HCCs [16]. Second, Raf kinase over-expression occurs in most HCCs. For example, in a study on HCC tissue specimens, the *Raf-1 *gene was up-regulated in 50% of 22 HCC specimens and activated Raf-1 protein was over-expressed in 100% of 30 HCC specimens [17]. Third, a variety of upstream growth factors, such as epidermal growth factor, VEGF, platelet-derived growth factor-β and transforming growth factor-α, which are generally over-expressed in HCC, can activate this pathway through binding their receptor tyrosine kinases [8,18].

The pERK protein is a key downstream component of the MEK/ERK cascade. In this study, basal levels of pERK were determined by immunocytochemical analysis and western blot analysis in order to evaluate the activation of the RAF/MEK/ERK pathway in four types of HCC cell lines with different metastatic potential. The results revealed that basal pERK levels increased stepwise in cell lines in accordance with their metastatic potential, indicating that the RAF/MEK/ERK pathway may be involved in tumor invasion and metastasis in HCC, consistent with results of previous studies [19].

Sorafenib is a multikinase inhibitor that inhibits the Raf serine-threonine kinases and blocks the RAF/MEK/ERK signaling pathway. Changes in the levels of pERK protein were determined by immunocytochemical analysis in these HCC tumor cells. Sorafenib inhibited ERK phosphorylation in a dose-dependent manner between 5 and 20 μM. However, further analyses revealed that the degree of inhibition in these HCC cell lines was significantly different according to their basal pERK expression levels. We found that the sorafenib pERK inhibition effect in SMMC-7721 cells, with lower pERK levels, was significantly weaker than the other three HCC cell lines with relatively higher basal pERK levels. On the contrary, no significant change in pERK phosphorylation was observed after 5-FU treatment.

It is possible that the antitumor activity of sorafenib might be due to its ability to inhibit angiogenesis-related tyrosine kinases as well as other RAF/MEK/ERK-independent mechanisms. For example, Raf-1 has been proposed to induce the phosphorylation of proteins that control apoptosis independently of MEK and ERK [15]. Additionally, the results of clinical trial analyses of sorafenib in renal cell carcinoma and melanoma have not provided sufficient information to conclude that the clinical value is associated with inhibition of the RAF/MEK/ERK signaling pathway [8].

However, positive results were observed in this study. In cell viability assays, sorafenib inhibited proliferation of all HCC cell lines with different basal pERK levels in a dose-dependent manner. Furthermore, the effects of sorafenib were significantly correlated with basal pERK levels in these HCC cell lines by correlation analysis between the IC_50 _values of sorafenib and their pERK density values, indicating that sorafenib sensitivity could have direct links with the activation of the RAF/MEK/ERK signaling pathway and basal pERK levels in HCC tumor cells.

To more directly determine the relationship between pERK expression and sensitivity to sorafenib, we used U0126, a selective inhibitor of MEK 1/2, to inhibit the MEK/ERK pathway and reduce basal pERK expression in MHCC97-H cells without influencing cell proliferation. We then assessed cellular responsiveness to sorafenib after pERK down-regulation. The observations showed that the pre-treated cells expressed much lower levels of pERK and became significantly less sensitive to sorafenib-mediated growth inhibition. These observations are perfectly consistent with our hypothesis that the RAF/MEK/ERK signaling pathway is essential for sorafenib-mediated growth inhibition and that sensitivity to sorafenib is directly related to the activation of this pathway and basal pERK expression. These results also confirm the findings of Ghassan K Abou-Alfa's group in a phase II clinical trial on treating advanced HCC patients with sorafenib that found that patients with tumors containing higher levels of pERK were more sensitive, or responsive, to sorafenib, supporting the notion that pERK may be a useful biomarker in treating HCC with sorafenib [9]. In our opinion, this was probably because blocking of the RAF/MEK/ERK signaling pathway plays a central role in sorafenib antitumor activity in HCC cells. As a key downstream component of this pathway, pERK levels could reflect the constitutive activity status of this signaling pathway, as well as the degree of inhibition of this pathway by sorafenib. Our study provides further *in vitro *evidence that pERK could be a useful biomarker predictive of a response to sorafenib in HCC tumor cells.

Resistance to the traditional chemotherapy drug 5-FU was closely associated with basal pERK expression in these HCC cell lines. Thus, the RAF/MEK/ERK pathway may be involved in the development of drug resistance to traditional chemotherapy in HCC, as reported in previous studies in other types of cancer. For instance, in breast cancer cells, activated Raf conferred resistance to the chemotherapeutic drugs doxorubicin and paclitaxel by inducing the expression of the drug pump Mdr-1 and the Bcl-2 antiapoptotic protein [15]. The results reported here would provide clues for further studies on reducing drug resistance by blocking the RAF/MEK/ERK signaling pathway and rationally combining sorafenib with other traditional cytotoxic agents to further improve efficacy.

## Conclusion

These experiments demonstrate that the RAF/MEK/ERK pathway might be involved in drug resistance to traditional chemotherapy in HCC cell lines. More importantly, our study provides further *in vitro *evidence that pERK could be a useful biomarker predictive of sensitivity to sorafenib in HCC tumor cells.

## Abbreviations

DMEM: Dulbecco's modified Eagle's medium; DMSO: dimethyl sulfoxide; ERK: extracellular signal-regulated kinase; 5-FU: 5-fluorouracil; HCC: hepatocellular carcinoma; MEK: mitogen-activated protein kinase kinase; pERK: phosphorylated ERK; VEGF: vascular endothelial growth factor.

## Competing interests

The authors declare that they have no competing interests.

## Authors' contributions

ZZ was primarily involved and responsible for work related to immunocytochemistry and drafted the manuscript. XZ carried out or supervised all work related to cell culture and drug administration. HS participated in the immunoblot analysis. DW participated in the statistical analysis. YW conceived the study and participated in its design and coordination. All authors were involved in drafting the manuscript and all approved the final manuscript.

## Pre-publication history

The pre-publication history for this paper can be accessed here:


